# Analysis of Individual Viral Particles by Flow Virometry

**DOI:** 10.3390/v16050802

**Published:** 2024-05-18

**Authors:** Caroline O. Tabler, John C. Tilton

**Affiliations:** Center for Proteomics and Bioinformatics, Department of Nutrition, School of Medicine, Case Western Reserve University, Cleveland, OH 44106, USA; cot3@case.edu

**Keywords:** flow virometry, nanoscale flow cytometry, viral quantification, viral sorting

## Abstract

This review focuses on the emerging field of flow virometry, the study and characterization of individual viral particles using flow cytometry instruments and protocols optimized for the detection of nanoscale events. Flow virometry faces considerable technical challenges including minimal light scattering by small viruses that complicates detection, coincidental detection of multiple small particles due to their high concentrations, and challenges with sample preparation including the inability to easily “wash” samples to remove unbound fluorescent antibodies. We will discuss how the field has overcome these challenges to reveal novel insights into viral biology.

## 1. Introduction

Flow cytometry is an invaluable biological tool for the high-throughput analysis of individual cells. By fluorescently labeling specific markers of interest, dozens of cellular attributes can be detected simultaneously. Not only can the presence of intracellular and membrane proteins be monitored, but many cellular processes including enzymatic reactions can be assessed.

Cytometry-based research has primarily been limited to the study of cells, and submicron particles have generally been understudied due to the various technological limitations that come with analyzing small particles. However, recent advances have led to a surge in flow cytometry-based assays that are specific to the study of nanoparticles, giving rise to the field of nanoscale flow cytometry (NFC). With NFC, the advantages of flow cytometry can be used to characterize small particles. The use of NFC to specifically characterize viruses is often referred to as flow virometry. In this review, some of the special considerations for adapting assays for flow virometry will be discussed, and many of the critical discoveries that have resulted from this technology will be highlighted.

## 2. Principles of Flow Cytometry

Flow cytometry allows for the high-throughput analysis of individual cells in a heterogeneous population. What began in the 1930s as a way to count cells is now an invaluable tool for the study of cellular biology [1]. As reviewed by Cossarizza et al. (2017), flow cytometers work by organizing cells into a single file line and using lasers to characterize individual cells [2]. Hydrodynamic focusing is typically used to align cells for single-cell analysis. A sheath fluid, typically saline, is pumped through a tube to create a laminar flow, and the cellular sample is injected directly into the center. As the tube diameter decreases, the sample stream diameter also decreases, which forces the cells into a narrow single-file line. The cells are then individually passed in front of several lasers for characterization of size and fluorescence.

Information about the size and shape of the cell is garnered from how much light the cell scatters. Forward scattering (FSC) varies based on the cell’s size and is typically measured based on the laser light scattered up to a 20-degree angle. Side scattering (SSC) is dependent on the internal complexity of the cell and is often measured at a 90-degree angle. In addition, lasers of various wavelengths are used to detect the presence of different fluorescent markers. Modern cytometers typically have up to seven lasers with wavelengths between 325 and 650 nm that can excite several fluorescent labels at once. Following excitation, the cellular fluorescence is channeled through numerous filters that separate the emitted light based on its wavelength. This allows the cytometer to detect multiple fluorescent compounds that were excited by the same laser but emit light at different wavelengths. The light is collected by photomultiplier tubes or photodiodes and quantified by analog to digital converters connected to a computer. Significantly, thousands of cells can be analyzed every second, permitting the acquisition of huge amounts of data in a short period of time.

## 3. Technical Considerations for NFC Analysis

As the name suggests, flow cytometers were originally designed to analyze cells, but they are increasingly being used to study much smaller particles. Flow cytometry was first used to detect bacteriophages in 1979 using a custom instrument with a small interrogation volume designed by Howard Shapiro and colleagues [3], although detecting viruses using commercial instruments remained challenging. In 2013, Arakelyan and colleagues reinvigorated the field of nanoscale flow cytometry by demonstrating that individual HIV-1 particles could be probed for membrane-bound antigens, and additional improvements in instrumentation and methodology have enabled the interrogation of viruses using commercial instruments [4,5,6]. Unlike Western blot analysis, nanoscale flow cytometry can distinguish between and analyze individual virions in a high-throughput manner. Although not a trivial assay, the continual development of highly sensitive flow cytometers has led to an increase in nanoscale flow cytometry being used to study viral characteristics, including particle size and surface protein expression.

**Figure 1:**
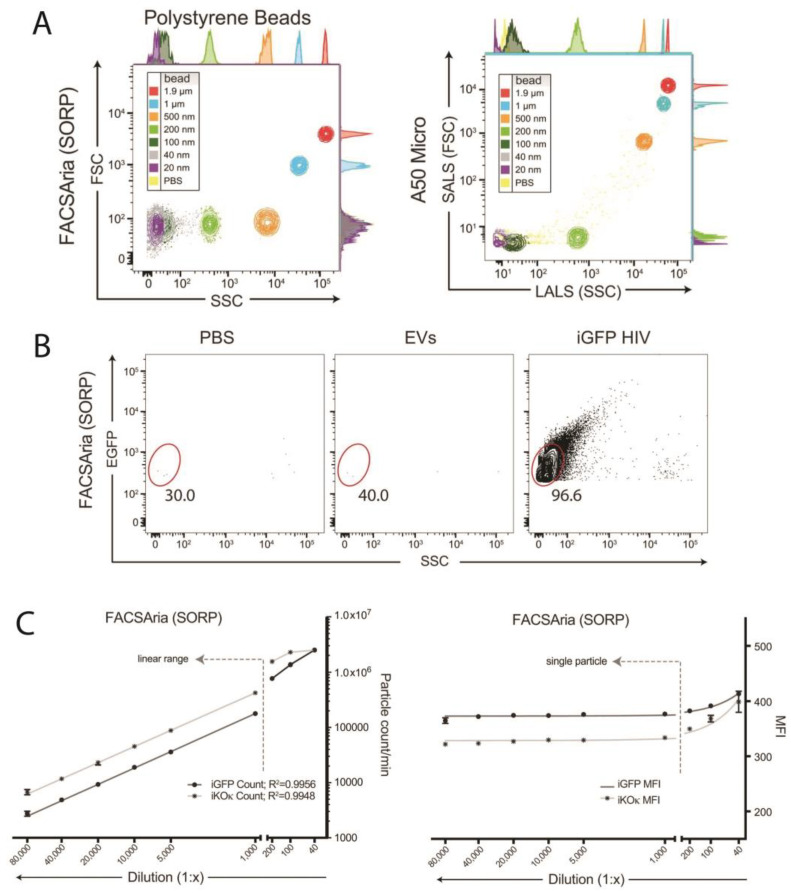
Detection of small particles and HIV-1 using flow cytometry. (**A**) Analysis of polystyrene beads of defined sizes by FSC and SSC properties on the FACSAria II (SORP) and A50 Micro flow cytometers [12]. (**B**) Analysis on the FACSAria II of PBS, untransfected HEK293T supernatants, or supernatants from cells transfected with a ΔEnv Gag-iGFP core plasmid (HIV-1 with EGFP cloned into the Gag polyprotein). Red gates are drawn on EGFP+ events with minimal light scattering, indicative of small fluorescent virions [12]. (**C**) Particle count/min and the mean fluorescent intensity (MFI) on the FACSAria II of iGFP and iKOκ viruses (HIV-1 with EGFP or Kusabira-Orange-κ cloned into the Gag polyprotein) by dilution factor on the FACSAria II (*p* < 0.0001 for both). All samples were measured for 1 min. Figure adapted with permission from Bonar et al. [12].

There are many challenges when using flow cytometers to study viruses, often referred to as flow virometry. Most cytometers are limited in the size of particles that they can accurately analyze. Cytometers are designed to record relatively large cells and disregard smaller events, including viruses, extracellular vesicles (EVs), and small particulate contaminants. It is standard procedure for cytometers to have a minimum threshold set using the size parameter FSC to prevent particles less than 300 nm in diameter from being recorded. The light scatter threshold value can be modified to increase the detection of small particles, but even at the lowest threshold setting, viruses and EVs smaller than 100 nm are generally indistinguishable from the cytometer internal background noise (Figure 1A). However, newer flow cytometer models with improved sensitivity, including Beckman Coulter’s CytoFLEX nano, have potential resolutions as low as 40 nm based on the detection of latex polystyrene beads [7].

Cytometers can be adapted in several ways to increase the nanoparticle signal-to-noise ratio. High-power and short-wavelength violet lasers can improve the light refraction from small spherical particles and assist in their detection [8]. Nanoparticles are also detected better using SSC rather than FSC [9]. In addition, the removal of small contaminants by stringent filtering of the cytometer sheath fluid and the purification of samples using methods like tangential flow filtration and size exclusion chromatography can increase the sensitivity of NFC [4,10,11]. To confirm that detected events are actual lipid-containing particles and not contaminants, samples can also be treated with a detergent like Triton X-100, which will dissolve membranes but leave contaminants intact.

While analyzing small particles using light scatter alone can be useful with a purified sample, it is still challenging to characterize heterogeneous populations. An alternative approach to detecting small particles is to fluorescently label the particles and apply a threshold based on fluorescence instead of light scattering, such that only fluorescently labeled particles are recorded regardless of their size (Figure 1B). The fluorescent channel voltage can then be modified to optimize the cutoff between fluorescent particles and non-fluorescent noise. Several approaches for labeling particles have been successfully used for NFC, which will be described in the following section.

The power of cytometers resides in their ability to individually analyze events, but nanoparticle samples are often much more concentrated than cells, and precautions must be taken to ensure single particle detection when performing NFC. If the sample concentration is too high, multiple particles can pass in front of the cytometer laser at the same time and be recorded together as a single data point, an issue known as coincidental or swarm detection [13]. It is important for investigators to perform a dilution analysis of their sample to ensure they are in the linear range of the assay, where event count is proportional to the dilution and the mean fluorescent intensity (MFI) of recorded events is stable (Figure 1C). This indicates that each event represents a single submicron particle such as an EV or virus, as multiple fluorescent particles recorded as a single flow cytometry event would have an increased MFI.

NFC can be a highly sensitive assay, but it is also prone to artifacts caused by reagent and equipment variability. This is especially true when trying to reproduce results across laboratories. MIFlowCyt-EV stands for “Minimum Information about a Flow Cytometry experiment in an EV-specific reporting framework” [14]. This article created standard guidelines for NFC experimental design, sample preparation, assay controls, instrument calibration, and data acquisition. MIFlowCyt-EV guidelines are instrumental in ensuring experimental reproducibility and protecting against misinterpretation of results. NFC is a rapidly developing field still in its early stages and pushes the boundaries of the optical systems of flow cytometers. Minor changes in instrumentation or protocol can lead to significant variability [15,16], highlighting the importance of developing standards and controls to make NFC experiments comparable across laboratories. We anticipate these guidelines will continue to evolve as experiments become more complex and established standardization methods are adopted. Currently, it is important to confirm results obtained from NFC using alternative, more established methods. 

## 4. Methods to Fluorescently Label Viruses

### 4.1. Lipid and Cytosolic Dyes

There are several nonspecific approaches for labeling cells that have been adapted for flow virometry. Lipophilic membrane dyes, such as PKH26 and DiD, have been used to label both EVs and viruses [17,18]. This allows virions to be tracked using fluorescence, and parameters such as concentration can be assessed using flow virometry. Producer cells containing these dyes may naturally pass them on to nascent viruses and EVs, or purified particles may be labeled with dye directly [19]. However, the use of many lipophilic dyes has been criticized for its tendency to produce artifacts. Lipophilic dyes may inadvertently label contaminants such as lipoproteins, and many of these dyes are capable of forming micelles, which can be mistaken for virions when analyzing them by NFC [11]. Membrane labeling has also been reported to increase the size of small particles, which could alter the results of subsequent analyses [11]. However, novel lipophilic dyes have been developed specifically for NFC that overcome these limitations [19]. Conjugated oligoelectrolytes were found to label EV membranes without aggregating and with a wide possible range of spectra [19].

Cytoplasmic dyes such as CFSE can also be used to label small particles, which appear less prone to aggregation when compared to other lipophilic dyes [11]. However, CFSE requires processing by esterases and therefore may not be suitable for all particles [20]. As with many methods to label small particles, the removal of unbound dye prior to analysis, for example with size exclusion chromatography, can increase sensitivity when performing flow virometry [4,10,11,19]. Importantly, none of these methods are capable of exclusively and specifically labeling all virions, and the labeling efficiency can vary based on the producer cell type. Furthermore, if there is a specific population of interest, more precise methods of labeling are required.

### 4.2. Genomic Labeling

Genomic labeling is an additional strategy for fluorescently tagging viruses containing nucleic acids. Both RNA and DNA stains have been used to visualize small particles, although the efficacy of individual dyes can vary. The SYTO line of nucleic acid dyes is common, and they have been successfully used to identify viruses, bacteria, and EVs using NFC [21,22,23]. While genomic staining may be slightly more specific than membrane labeling in terms of identifying intact virions, discriminating between different subsets of nanoparticles likely requires additional labeling of proteins [24].

### 4.3. Protein Labeling

Labeling specific proteins allows for a more precise analysis of subsets within a heterogeneous sample. However, care must be taken when working with antibodies, as unbound antibody aggregates may need to be removed from the samples to prevent them from being recorded as events. This can be accomplished in several ways including by purifying cells through either immunomagnetic separation, ultracentrifugation, or size exclusion chromatography, centrifuging antibodies before use, or combining a lower antibody concentration with longer staining times [4,11,25,26].

Of course, these strategies require that the labeled proteins are abundant enough to produce a sufficient fluorescent signal, which can be challenging with viruses incorporating limited protein. When using NFC to study viruses, the viruses themselves should be specifically labeled to distinguish them from the similarly sized EVs that contaminate viral samples [24]. This can be challenging given that EVs will be present in all viral preparations and are capable of packaging viral proteins [27]. Therefore, proper controls should be designed to determine if viruses, EVs, or a mixture of both are being detected. When studying viruses, it is also worthwhile to consider assessing EVs, which can play a biologically relevant role in viral pathogenesis [27]. 

Because the number of proteins in viruses is relatively low, it can be beneficial to use antibodies with bright fluorophores like PE (Phycoerythrin) [6,28]. Viral proteins can be specifically labeled not only with antibodies but also through the incorporation of a fluorescent protein tag. However, it should be ensured that the tag does not alter virion function [29,30]. In addition, antibodies can be used to conjugate particles of interest to beads. By binding particles to beads, their apparent size is increased, which can facilitate their detection by NFC [29]. Using magnetic beads, specific subsets of virions can be purified prior to analysis, and the virions can then be further probed with additional antibodies, providing complex information about heterogeneity within viral populations [4,18].

## 5. Current Applications of Flow Virometry

Methods to characterize virions often rely on the indirect bulk analysis of samples. A large population of viruses must be analyzed at the same time to obtain data from assays like ELISAs and Western blots, which masks heterogeneity at the per-particle level. However, the emergence of flow virometry has provided an opportunity to assess individual virions and quantify their expression of various markers. In addition, flow virometry has a higher sensitivity than many other assays, with a much smaller sample size required to quantify and study small particles. With flow virometry, not only can far fewer viruses be used to determine concentration, but a much more direct estimation of viral concentration is possible through the detection of individual intact viruses. 

### 5.1. Particle Concentration

Many of the initial applications of flow virometry involved particle quantification [31,32]. In many instances, particle quantification makes use of fluorescently labeled particles, and therefore reliable labeling is a necessity to trust concentration measurements. Generating a standard curve using samples of known concentration can assist in accurately determining absolute concentrations. This is easier than ever with the commercial availability of several standards, including beads as well as more biologically relevant lipid nanoparticles [16,33,34].

When applied correctly, flow virometry is unparalleled in the speed and sensitivity with which small particles can be detected, making flow virometry competitive with other methods for quantifying particles. We found that the detection of fluorescent HIV-1 by flow virometry has a sensitivity approaching PCR-based assays, with a limit of detection of around 80 particles/mL [12].

### 5.2. Particle Size

The first report of flow virometry used light scattering to differentiate bacteriophage T2 from reovirus based on size [3]. More recently, Junin viruses as small as 40 nm and three distinctly sized populations of vaccinia virus were distinguished using flow virometry [35,36]. A specialized ultrasensitive cytometer with a resolution of 27 nm even allowed the discrimination of intact bacteriophage virions from their empty capsids and naked genome [37]. 

**Figure 2:**
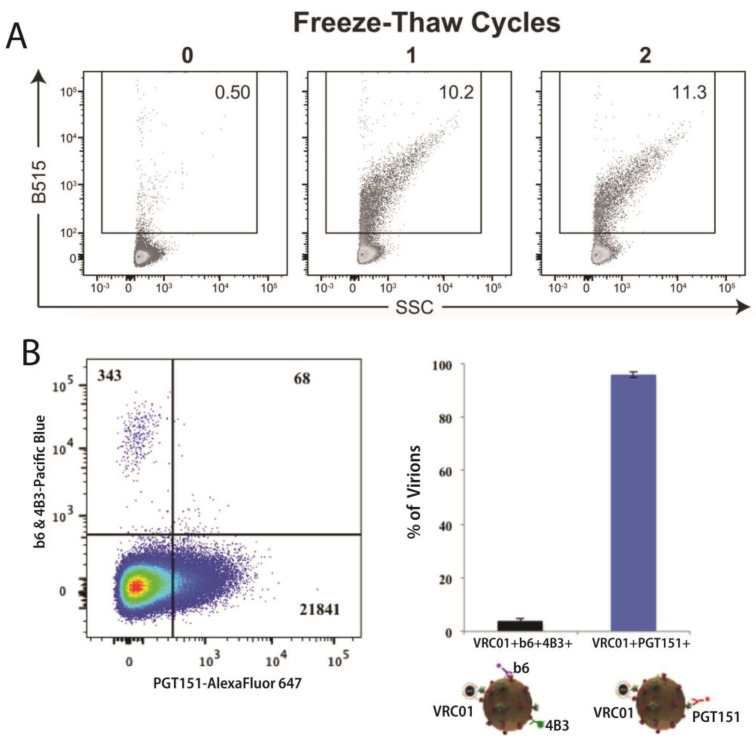
Applications of flow virometry. (**A**) EGFP-labeled HIV-1 viral particles prior to and after freeze–thaw cycles using particles bearing an R5-tropic HIV-1 Env. The numbers above each plot indicate freeze-thaw cycles. The viral aggregates are gated based on increased size (SSC) and fluorescence (B515-EGFP). Figure adapted with permission from Bonar et al. [12]. (**B**) Flow virometry of T/F HIV-1 CH162 virions. Distribution of anti-Env VRC01-magnetic nanoparticle-captured virions stained with three anti-Env antibodies: AlexaFluor 647-labeled PGT151, Pacific Blue-labeled b6 and 4B3. Viruses that bound to both VRCO1 and PGT151 (specific for mature, processed Env) did not efficiently bind to b6 or 4B3 (specific for immature and defective Env). A pseudocolor plot displays event density detected by flow virometry, with blue and red corresponding to areas of lowest and highest cell density, respectively. The bar graph summarizes the mean ± SEM of three experiments. Adapted via a Creative Commons Attribution 4.0 International License (https://creativecommons.org/licenses/by/4.0/, accessed on 29 April 2023) from Arakelyan et al., Figure 6 [39].

Viral particles can also change size in response to environmental conditions. Light scattering revealed that influenza A changed shape in the presence of antiviral antibodies, suggesting that different virion shapes may influence immune escape [38]. In addition, the effect of factors like filtration, centrifugation, temperature, and freeze–thawing on the propensity for vaccinia virus and HIV-1 to aggregate was characterized by flow virometry (Figure 2A) [12,36].

However, the standardization of particle size remains difficult because reference beads have a higher refractive index than lipid particles, meaning beads and particles of the same size will scatter light differently. Recent software developments have made it easier to estimate particle size. FCM_PASS_, currently in version 4.1.1 released June 2022, makes use of commercially available reference beads to conform to the MIFlowCyt-EV reporting framework and standardize particle size and fluorescence measurements across laboratories [14,40]. More recently, lipid particles have become commercially available for NFC standardization and can overcome differences in refractive index [33,34].

### 5.3. Protein Expression

Flow virometry has increasingly been used to determine the presence and abundance of specific proteins in individual virions. Protein-specific antibodies and fluorescent protein tags have allowed an exploration of the heterogeneity within viral populations. As with particle size, the FCM_PASS_ V4.1.1 software and the appropriate reference materials will allow the standardization of fluorescence intensity corresponding to protein expression [40].

The variability of Herpes simplex virus (HSV-1) viral tegument and capsid expression was studied using a variety of fluorescently tagged viral proteins [23]. In addition, plasma-derived HIV-1 particles were captured with anti-Env-coated magnetic beads, and the heterogeneity of HLA-DR and LFA-1 expression on purified viruses was assessed using antibodies [4]. In a similar study, HIV-1 from patient plasma was again captured on fluorescent magnetic beads, and a correlation was found between CD36 and TGFβ expression on macrophage-derived HIV-1 [41].

Protein expression on virions can also provide information about the composition and source of the viral membrane. For example, Junin virions were probed with tetraspanins to demonstrate they originated from lipid rafts [35]. Similarly, murine leukemia virions were observed to bud from lipid rafts in a manner dependent on the expression of the accessory protein GlycoGag [42]. Showcasing the utility of flow virometry for high-throughput screens, protein expression on the HIV-1 membrane was recently tested using a panel of fluorescent antibodies, with several proteins including CD38 identified as novel virion markers [6]. 

### 5.4. Protein Conformation

In addition to identifying the presence of different proteins, flow virometry has been used to provide valuable information concerning differences in protein conformation. There have been several studies characterizing the fusogenic HIV-1 envelope (Env) protein using antibodies specific for functional and nonfunctional Env epitopes. Immunomagnetic capture combined with flow virometry allowed up to three antibodies to be assessed simultaneously on the same virion (Figure 2B) [39]. In this manner, it was determined that most viruses exclusively carry either a functional trimeric or a defective Env, suggesting that functional virions are protected from non-neutralizing antibodies produced in response to defective virions [39]. Another study found that expression of CD4, SERINC5, or Nef could alter the Env conformation on both HIV-1 virions and on cells [43]. HIV-1 envelope conformation was also dependent on the producer cell, as CD36+ macrophage-derived virions appeared to preferentially bind the broadly neutralizing antibodies 2G12/PG9 [41].

Conformational changes in response to viral maturation and fusion have also been analyzed. Dengue viral particles were stained with an antibody that binds the unprocessed prM membrane protein on immature virions [18]. After the immunomagnetic separation of virions, differences in viral maturation could be determined. In another study of the Nipah virus, antibodies that detected different conformations of the viral G envelope protein enabled the detection of G conformational changes in response to binding its receptor ephrinB2 [25].

### 5.5. Vaccine Quality Control

Flow virometry provides a unique opportunity to rapidly assess the quality of vaccines during production. The nucleic acid dye SYTO 82 and light scattering were used to discriminate intact virions from contaminants and to estimate the concentration and purity of veterinary vaccines [37]. Similarly, human cytomegalovirus vaccine particles were analyzed for purity and functionality after staining for the presence of both DNA and viral antigen [21].

Several studies have used flow virometry to detect the conformational changes of viral vaccines caused by damage. The Ebola virus vaccine ERVEBO was found to increase in size based on light scatter in response to increasing temperatures [44]. As previously mentioned, the vaccinia virus was prone to aggregation in response to factors like centrifugation and freeze–thawing [36]. By monitoring viral vaccine production using flow virometry, the preparation and storage conditions of vaccines can be optimized to ensure that issues resulting in viral damage can be quickly detected and rectified.

### 5.6. Enzymatic Activity

As flow virometry assays have evolved, it has become possible to monitor increasingly complex biological functions within virions, including protease activity. Our group has recently developed a reporter for HIV-1 protease activity referred to as VIPER (VIral ProteasE Reporter) [45]. VIPER is a FRET-based reporter containing a fluorescent mUKG (mUmikinoko-Green) and mKOκ (mKusabira-Orange-κ) FRET pair linked by a protease cleavage sequence. FRET, or Förster resonance energy transfer, occurs between two fluorescent proteins that are in close proximity. When VIPER is processed by protease, there is a relative loss of FRET and an increase in mUKG fluorescence that can be detected with a flow cytometer. VIPER is conjugated to the viral protein Vpr, which non-covalently associates with the HIV-1 Gag p6 protein and ensures VIPER is incorporated into budding virions [46]. 

Our assay provides a novel system to monitor proteolytic activity within intact virions. We found that the processing activity detected using flow virometry was comparable to that detected by Western blot [12]. We were also able to illustrate the heterogeneity of protease activity in various patient-derived samples and to identify viruses with resistance to HIV-1 protease inhibitors [45]. In addition, the cleavage sequence within the reporter can be modified, making it possible to differentiate the activity of protease when it is in different states of maturation [47]. In upcoming work, we will use VIPER-Vpr to characterize the impact of HIV-1 protease polymorphisms and resistance mutations. 

### 5.7. Sorting

The sorting of viral subsets can provide a great deal of insight into their functional biology. Sorting using flow virometry allowed the infectious HSV-1 C-capsid to be independently isolated and studied based on its incorporation of a SYTO-labeled viral genome, which is lacking from non-infectious capsids [22,48]. Similarly, HIV-1 was labeled with broadly neutralizing antibodies and sorted directly from infected patient plasma for sequencing and mass spectrometry analysis, although only a limited number of peptides could be detected due to the small sample size [49]. Vaccinia virus was also sorted by size and analyzed by droplet digital PCR to show that larger particles had increased copies of viral genomic DNA [36].

An important consideration for functionally characterizing viral subsets post-sorting is whether they remain infectious. Our group found that GFP-tagged HIV-1 particles could be sorted with no significant impact on viral infectivity [12]. Junin viruses labeled with a nucleic acid dye and antibody envelope also retained their infectivity post-sort [35]. Finally, HSV-1 particles were sorted based on the expression of GFP-tagged VP16 and VP22 tegument protein, and a correlation was found between increased tegument expression and viral infectivity [23]. 

## 6. Conclusions

As interest in analyzing small particles grows and more advanced instrumentation becomes available, performing flow virometry becomes easier every year. With this powerful tool, the unexplored heterogeneity within viral populations can be fully appreciated. Flow virometry makes it possible to specifically isolate and characterize virions based on specific properties, which was not feasible when performing bulk sample analysis. This technology combined with sorting could allow us to obtain unparalleled detail about what viral components impact infectivity, research that is already underway.

There are still many challenges that must be overcome to realize the full power of flow virometry. Due to the limited cargo within small particles, it is still much more challenging to detect proteins in virions than it is in cells. Efforts to reduce the presence of auto-fluorescent contaminants through improved sample purification and the development of brighter labeling fluorophores may improve the detection of proteins in low quantity, which will be essential for analyzing increasingly specific subsets of virions. Improved standardization and calibration reagents to enable comparisons of experiments completed by different laboratories on different instruments are also a major need in the field.

In addition, it will be important to carefully define which markers can be used to accurately label various small particles for analysis by flow virometry. For example, virions can often be mistaken for EVs and vice versa, as virions can package many cellular proteins while EVs also package viral proteins [27]. For example, we found that EVs packaged the SARS-CoV-2 spike protein and specifically interacted with anti-spike antibodies [50]. Indeed, virally infected cells likely produce a heterogenous spectrum of small particles: EVs packaging no or only a few viral proteins, EVs containing higher levels of spike or other viral components, defective viruses, and infectious viruses that interact in complex ways with target cells and the immune system.

Flow virometry is a powerful technology for unraveling this heterogeneity and may lead to more effective vaccines and treatments for viral infections by defining the phenotypes of EVs and viruses, especially features of the infectious viruses that need to be neutralized. The ability to determine not only what proteins are present in virions but also their biological conformation is certainly not a trivial task. However, with the recent development of new reagents, instrumentation, and protocols and our increased awareness of engineering limitations, it appears within our reach to begin evaluating viruses with an incredible amount of rigor. As it becomes increasingly feasible to detect small particles using flow virometry, we can expand our understanding of the heterogeneity of viruses and how that variability impacts vaccination and pharmacologic treatment strategies.

## Data Availability

No new data was generated for this review.
